# Acetabular reinforcement ring with additional hook improves stability in three-dimensional finite element analyses of dysplastic hip arthroplasty

**DOI:** 10.1186/s13018-018-1023-7

**Published:** 2018-12-07

**Authors:** Koji Totoribe, Etsuo Chosa, Go Yamako, Xin Zhao, Koki Ouchi, Hiroaki Hamada, Gang Deng

**Affiliations:** 1 Department of Orthopaedic Surgery, Faculty of Medicine, University of Miyazaki, 5200 Kihara, Kiyotake, Miyazaki, 889-1692 Japan; 2 Department of Mechanical Design Systems, Faculty of Engineering, University of Miyazaki, 1-1 Gakuen Kibana-dai-Nishi, Miyazaki, 889-2192 Japan

**Keywords:** Finite element method, Total hip arthroplasty, Acetabular reinforcement ring, Primary implant stability

## Abstract

**Background:**

The stability of acetabulum reconstructions using reinforcement rings and hooks is important for successful replacement surgery. The objective of this study was to biomechanically determine the effects of the hook on stress and the related micromotions of the acetabular reinforcement ring during the immediate postoperative period.

**Methods:**

Acetabular reinforcement ring models were developed using a nonlinear, three-dimensional, finite element method. Using a pre-prepared template, we constructed without-hook and bone graft models of varying volumes and material properties.

**Results:**

The stress on the inferior margin of the acetabulum was higher in the with-hook model than in the without-hook model, especially with increased bone graft volumes, and the stiffness of the bone graft material was decreased. Relative micromotions in the without-hook model were higher than in the with-hook models. The highest relative micromotion was observed in the model with increased bone graft volume and lower stiffness of bone graft material.

**Conclusions:**

In biomechanical analyses, the hook effectively dispersed stress and improved the initial fixation strength of the acetabular reinforcement ring.

## Background

Total hip arthroplasty (THA) is a widely used replacement surgical method that requires primary implant stability. Under conditions of severe bone deficiency around the acetabulum, acetabular reconstructions are needed to provide bony support for acetabulum restoration [1–4]. To this end, acetabular support rings can address severe bone stock deficiencies in patients with acetabular dysplasia or revision THA. Ganz previously developed an acetabular reinforcement ring with a hook (ARRH) for bone grafts and demonstrated improved fixation of the grafted bone, initial fixation of the cup, and stability of the reconstructed acetabulum [5]. In these procedures, the hook of the ring is placed around the inferior margin of the acetabulum to facilitate the placement of the cup in the correct anatomical position. Although the effects of the hook have not been investigated in mechanical analyses, proper placement of the hook is believed to improve the primary stability of the ring and to prevent migration [6]. As migration of the ARRH with breakage of the hook indicates a loosening of the acetabular component [7–11], biomechanical analyses are increasingly performed using the finite element method, which has been used by several investigators to analyze load distributions of acetabular reinforcement rings [12–14]. We also reported the effects of the ARRH on acetabular dysplasia, and compared the efficacy of varying numbers and insertion sites of screws [15], but did not conduct detailed analyses of the biomechanical roles of the hook. In this study, we used the finite element method to develop a detailed model of the hip joint and analyzed the biomechanical effects of the hook of the ARRH in bone grafts for acetabular dysplasia. In these analyses, we considered the severity of acetabular dysplasia and the type of bone graft material used.

## Methods

**Fig. 1:**
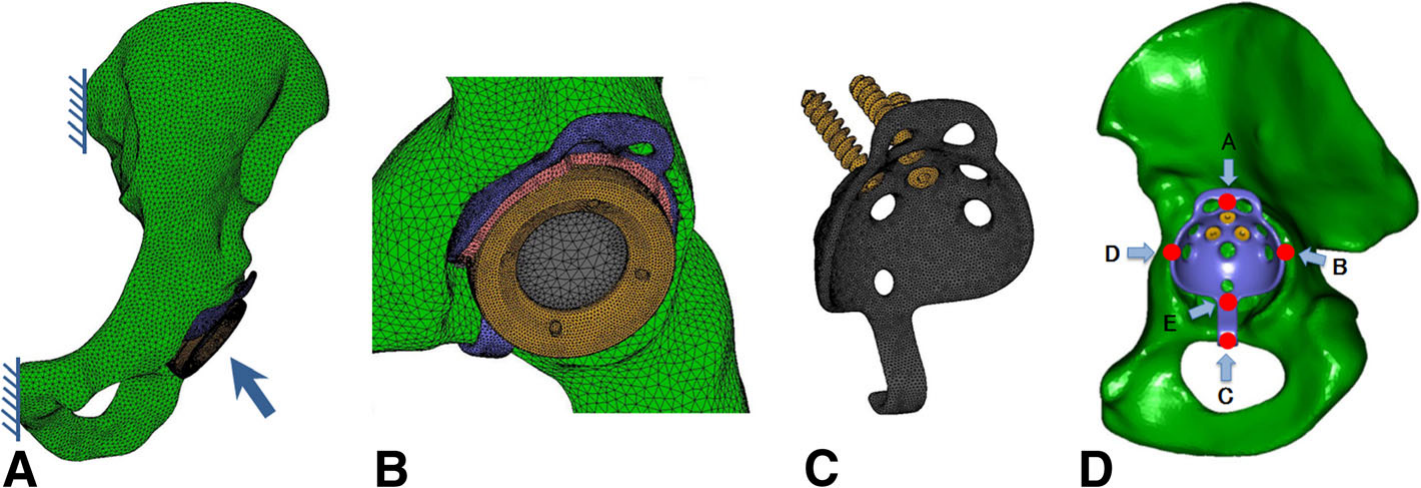
Three-dimensional total hip arthroplasty (THA) with the acetabular reinforcement ring with a hook (ARRH) model. **a** Shaded areas are fixed in all directions and the arrow indicates the loading point. **b** Magnified oblique view of the Ganz ring, the cement, the acetabular cup, and the femoral head. **c** ARRH with screws. **d** A, B, C, and D: measurement points for von Mises stresses on the pelvic bone around the ARRH; B, D, and E: measurement points for relative micromotion between the ARRH and the pelvic bone

To develop a finite element model, geometric data were obtained using computed tomography of the sawbone (Sawbones, Pacific Research Laboratories, Inc., Vashon, WA) left pelvic model at a slice thickness of 0.6 mm. A basic model of the left pelvic bone was constructed with total element and node numbers of 6043039 and 121231, respectively (Fig. 1). To develop these models and perform analyses, we used the multipurpose finite element analysis software MARC/Mentat (MSC Software Corp., Santa Ana, CA). The mesh of the cortical bone, the trabecular bone, the bone cement, the Ganz ring, and the prosthetic head for finite element models were reconstructed using four nodes of solid linear tetrahedral elements. In previous studies, Young’s moduli of these elements were 17,000, 100, 2100, 110,000, and 230,000 MPa, respectively, and corresponding Poisson’s ratios were 0.3, 0.2, 0.4, 0.3, and 0.3 [16–21]. In contrasting studies, Young’s moduli of morselized bone grafts were 42–150 MPa [22–24].

In the present bone graft models, we simulated the treatment of acetabular defects with morselized bone grafts, as described previously [25–27]. Young’s moduli for the bone graft site were set at 42 (low) or 150 MPa (high). The respective low- and high-stiffness bone graft values were set according to previous reports, and the corresponding Poisson ratio was 0.2.

**Fig. 2:**
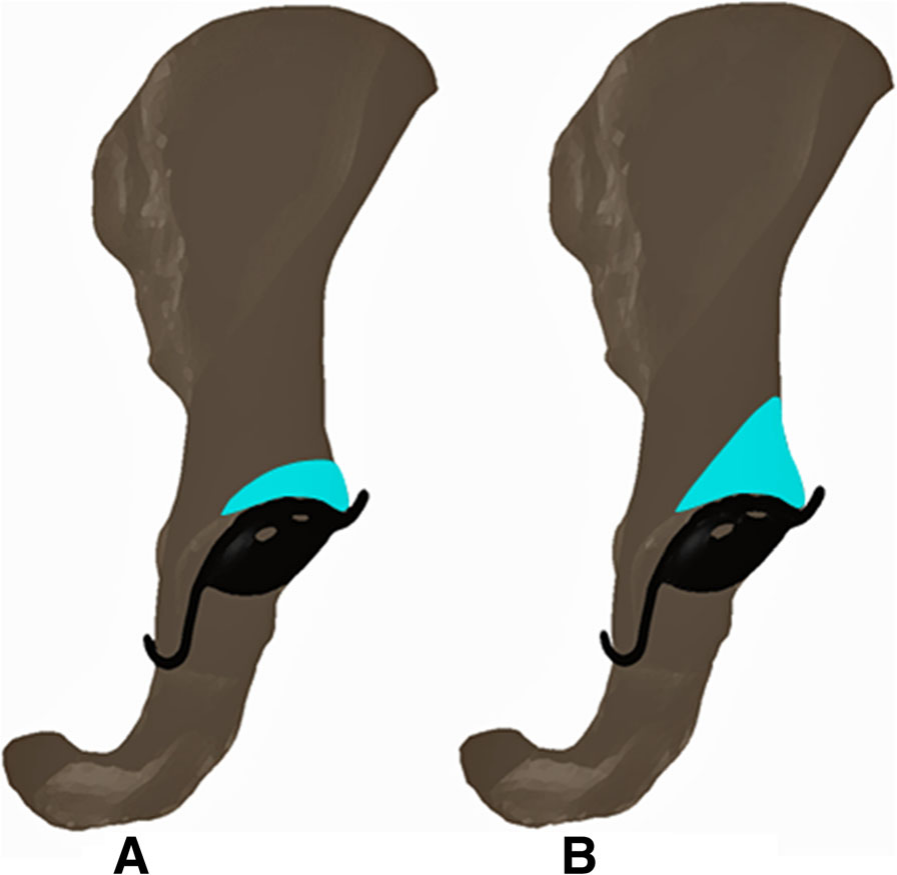
Post-bone grafting models for acetabular dysplasia: **a** type 1, 25% subluxation; **b** type 2, 62.5% subluxation

Crowe et al. [28] classified dysplastic hips into the following four groups according to degrees of subluxation: group 1, 0–50% subluxation; group 2, 50–75% subluxation; group 3, 75–100% subluxation; and group 4, dislocation. Herein, the following two acetabular dysplasia models (corresponding to groups 1 and 2) were developed based on the Crowe classification system: type 1, 25% subluxation and type 2, 62.5% subluxation (Fig. 2).

The ARRH (50 mm) was subsequently fixed to the acetabulum by inserting the hook into the obturator foramen and immobilizing the ring using three threaded 6.5-mm cancellous screws. The without-hook model was then created by omitting the hook element from the original ARRH. The interface between the ARRH and the pelvic bone was assumed to be a nonlinear contact problem, with friction set to 0.88 as described previously [29]. The screw–bone and screw–ring interfaces were assumed to be bound, and an acetabular cup (outer/inner diameters, 48/26 mm; MX Hip Joint Prosthesis, Mizuho Medical Inc., Tokyo, Japan) was mounted on the ring with a lateral opening angle of 45°, an anterior opening angle of 15°, and the assumption of 1-mm-thick cement fixation. The femoral head was modeled as a hemisphere and was bound to the cup.

Loading conditions were as described by Bergmann et al. [30]. In this model, force magnitudes and directions were normalized to the regular gait of patients with THA, and 1948 N was applied to the center of the head of each model. Regarding boundary conditions, areas corresponding to the sacroiliac joint and pubic symphysis of the pelvic bone were completely restrained. Nonlinear parametric analyses were performed with force control in the numerical procedure, and the Newton–Raphson method was used iteratively with incremental loads in 20 steps. After loading of models, von Mises stress distributions and relative micromotions were compared.

## Results

### Stress distributions

**Fig. 3:**
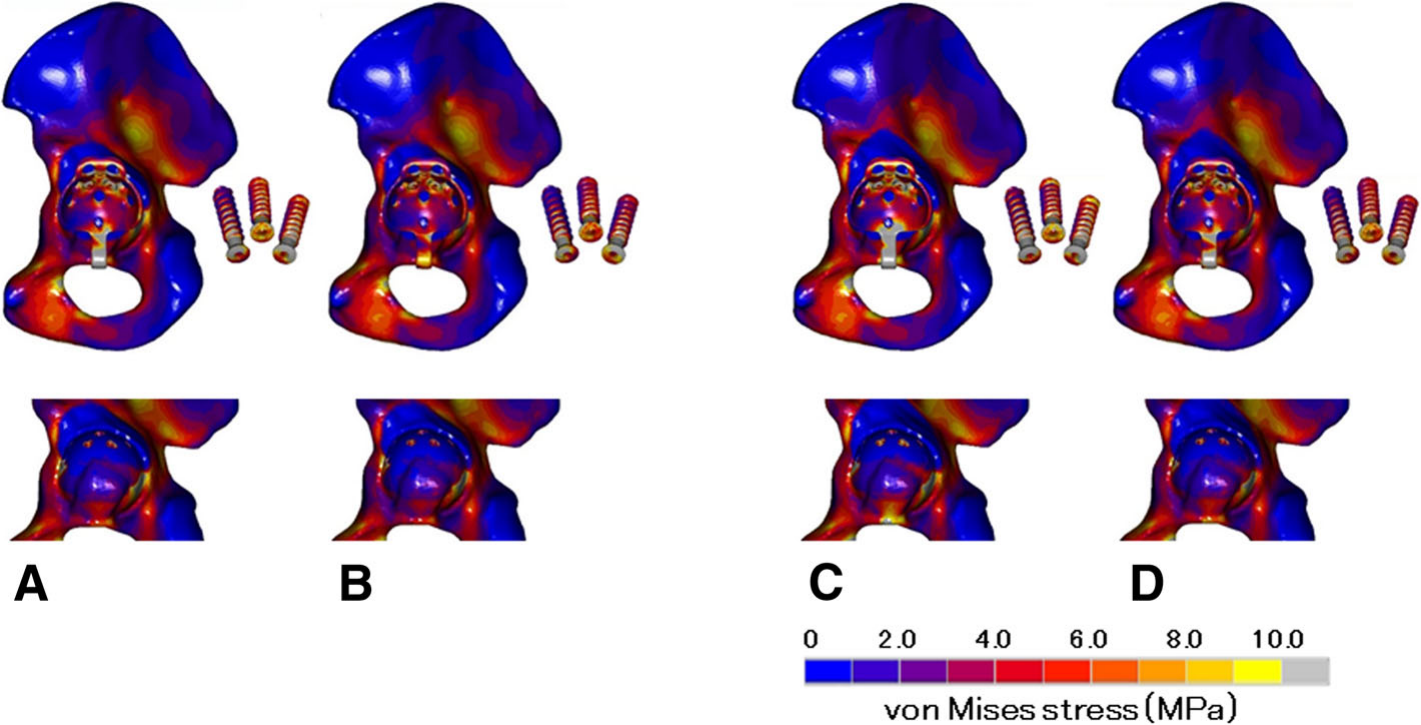
Distribution of von Mises stresses in with-hook models: top row, distribution of von Mises stresses in the pelvic bone, the Ganz ring, and the screws without an acetabular cup; bottom row, distribution of von Mises stresses in the acetabulum of each model without the ring; **a** type 1/low, **b** type 1/high, **c** type 2/low, **d** type 2/high

**Fig. 4:**
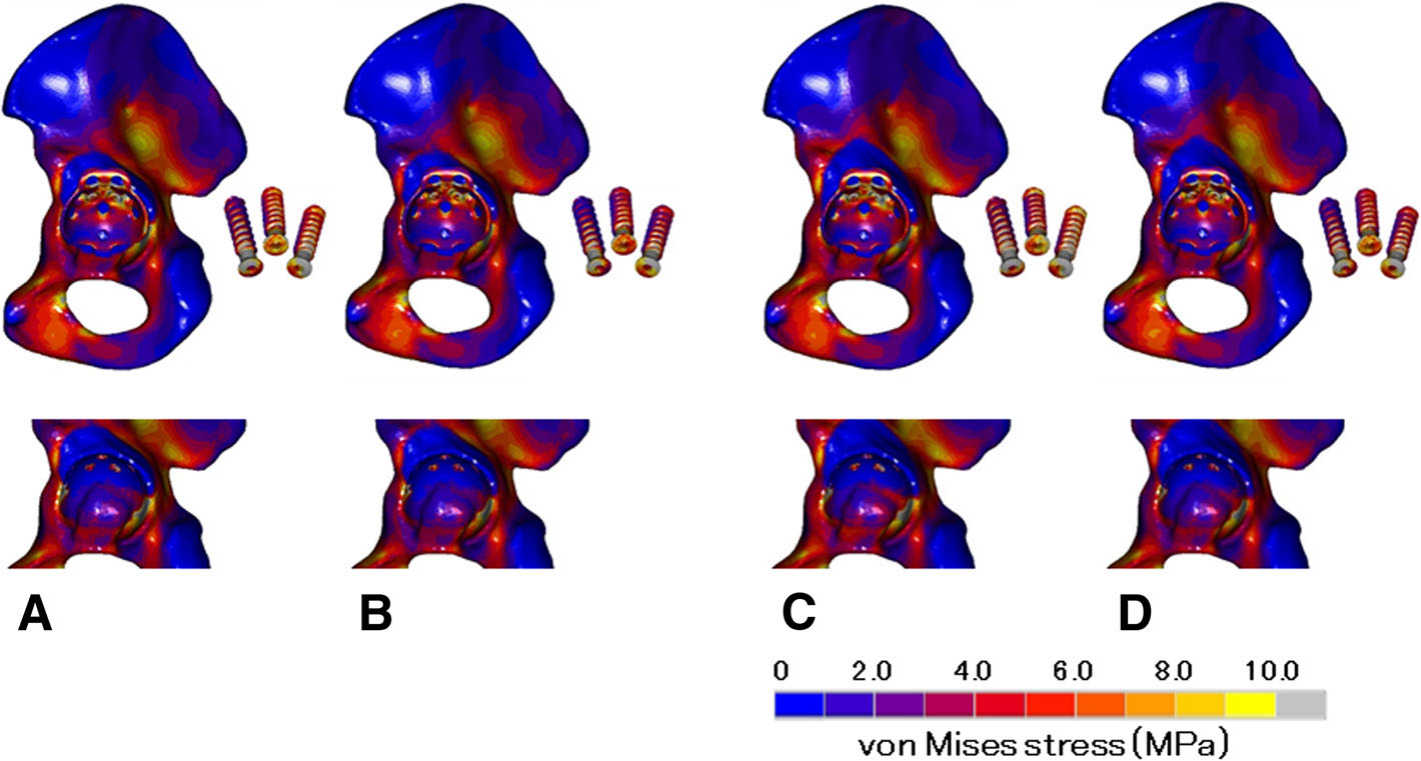
Distribution of von Mises stresses in without-hook models: top row, distribution of von Mises stresses in the pelvic bone, the Ganz ring, and the screws without an acetabular cup; bottom row, distribution of von Mises stresses in the acetabulum of each model without the ring; **a** type 1/low, **b** type 1/high, **c** type 2/low, **d** type 2/high

**Fig. 5:**
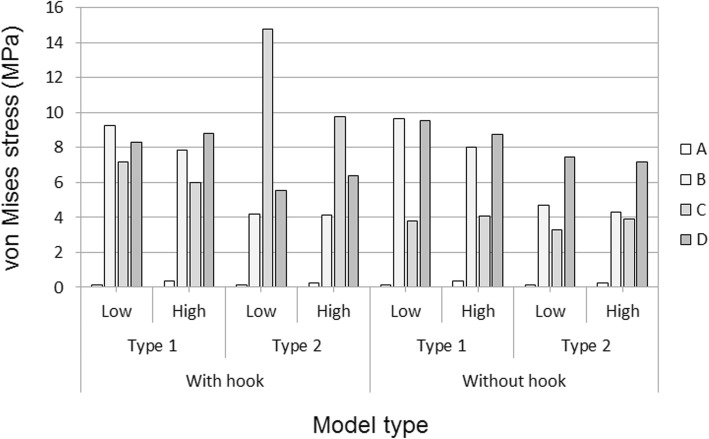
Maximum von Mises stress values at points A (upper part), B (posterior part), C (inferior margin part), and D (anterior part) of the ARRH

The ARRH with a hook showed higher von Mises stress values around the screw holes and the hook. Type 2 models showed higher stresses than type 1 models, and the low 42-MPa bone graft model showed higher stresses than the high 150-MPa bone graft model. In distributions of von Mises stresses in the acetabulum, lower and higher stress values were observed in and around the bone graft, respectively. Stresses at the inferior margin of the acetabulum, which is the contact area between the hook and the bone, were higher than in the without-hook model (Fig. 3), in which stresses at the inferior acetabulum host bone area were higher than in the with-hook model (Fig. 4). Von Mises stresses in the acetabulum were measured at multiple points around the ring and were lower at point A in the bone grafts of each model. In the with-hook model, higher stress values were observed at the hook contact area, which was at point C around the inferior margin of the acetabulum. The maximum von Mises stress value was observed in the type 2 model with the low bone graft (14.8 MPa), followed by that in the type 2 model with the high bone graft (9.8 MPa). In comparisons of with- and without-hook models, the stress at point C was lower and the stresses at points B and D were higher in the without-hook model (Fig. 5).

### Relative micromotions

**Fig. 6:**
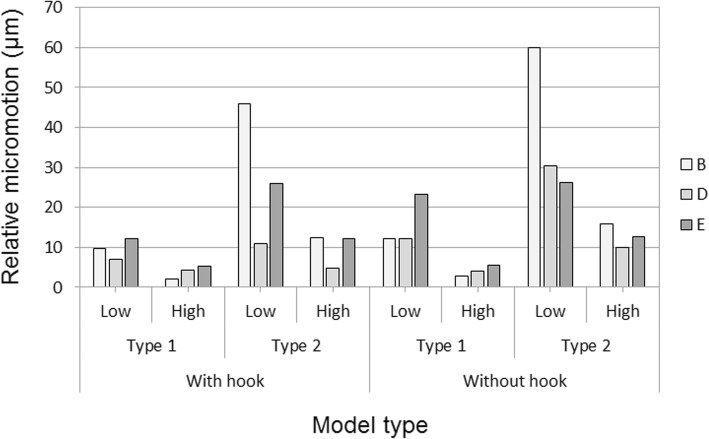
Relative micromotions at points B (posterior part), E (lower part), and D (anterior part) of the ARRH

The stability of the ARRH was evaluated according to relative micromotions, which were defined as distances between the ring and pelvic bone at the interface and were measured at points B, D, and E around the ring (Fig. 1d). Relative micromotions tended to be lower in the hook model than in the without-hook model. Specifically, in type 2/low models with the highest values, a 23% decrease from 60.0 μm was observed without the hook and this was only 46.1 μm in the with-hook model (Fig. 6).

Relative micromotions tended to be lower in type 1 than in type 2 models and tended to be lower with the high bone graft than with the low bone graft. Correspondingly, maximum micromotion values were highest in the type 2/low without-hook model and were lowest in the type 1/high with-hook model.

## Discussion

Herein, the effects of the ARRH hook were determined for various types of bone grafts. Our comparisons of with- and without-hook models confirm that the hook disperses stress and reduces relative micromotions. In the present models, the hook was inserted into the obturator foramen by immobilizing the ring in the anatomical position using three threaded cancellous screws. Following bone grafting with hooks, stresses on the bone graft were low, whereas those around the screw holes, screw, hook, and the contact area between the hook and the bone were high. Hence, the load in the morselized bone graft was dispersed to the normal bone around the acetabulum. In the inferior acetabulum host bone area, stress was higher in without-hook models than in with-hook models, probably reflecting the separate insertion of hooks and screws into the obturator foramen, and the good bone stock that protects the bone of the acetabulum. In contrast, rings were compressed into the acetabulum in without-hook models. Hook models also tended to have lower relative micromotion values than without-hook models. These stress and relative micromotion data suggest that the stress is dispersed biomechanically by the ARRH and that the hook can effectively disperse stress and improve the primary stability of the ARRH. In a previous clinical report by the ARRH, Gerber et al. [6] suggested that the primary stability of the implant is enhanced by adequate screw purchase in the ilium and proper placement of the hook below the teardrop, which pre-tensions the ring similar to that in plate osteosynthesis procedures that are performed using a tension device or blade-plate. Hassan et al. [10] also suggested that the oval geometry and the inferior hook of the ring contribute to the stability by preventing the rotation and migration of the components. Our results demonstrate the biomechanical role of the hook as described in the previous study, with enhanced primary stability of the implant in comparison to without-hook models.

In comparisons of bone graft sizes and elastic moduli of bone graft materials, relative micromotion values tended to increase with the severity of acetabular dysplasia and the stiffness of the bone graft decreased concomitantly, suggesting an increased risk of poor initial fixation in patients with severe bone stock deficiencies and low bone graft material properties. Clinically, it is important to achieve sufficient bone coverage and initial stability of the acetabular component, especially during the reconstruction of severe acetabular bone defects in patients with acetabular dysplasia and in patients with osteoporosis receiving revision THA. Several clinical studies report hook breakage with loosening and/or migration of the acetabular component [7–11]. Breakage of the hook or screw is recognized as a mechanical failure and reportedly occurs when the bone graft is not strong enough to support the ARRH [11]. Impaction during bone grafting was shown to increase the stiffness of the bone graft material and improve fixation and was applied during surgery [31–33]. In the hook model, the stress of the hook and screw in the type 2/low models was higher than in other models, suggesting an increased risk of poor initial fixation and mechanical failure. In patients with this type of massive bone defect, impaction bone grafting or bulk structural bone grafting are necessary to fix and to prevent mechanical failure [11, 34–36].

In this study, we investigated biomechanical aspects of the hook of acetabular reinforcement rings using finite element analyses. But we did not consider time-dependent mechanical responses, such as bone remodeling and ingrowth. As the severity of bone defects, the range of bone grafts, and the stiffness of bone grafts vary in clinical settings, the qualitative trends discussed here are to be applied with caution in clinical practice.

## Conclusions

We investigated the effects of the hook of acetabular reinforcement rings on stress distributions and relative micromotions between the ring and pelvic bone. In the present model of increased bone graft volume, we observed increased stress around the contact area between the hook and the bone and increased micromotion with lower stiffness of the bone graft material. The hook can effectively disperse the stress of the acetabular reinforcement ring and lead to greater fixation strengths. Thus, the appropriate spatial placement of the hook and the use of proper bone grafting techniques will result in better clinical outcomes, especially following the reconstruction of massive bone defects in patients with osteoporosis.

## Abbreviations

ARRH: Acetabular reinforcement ring with a hook
THA: Total hip arthroplasty
